# Lifetime health effects and medical costs of integrated stroke services - a non-randomized controlled cluster-trial based life table approach

**DOI:** 10.1186/1478-7547-8-21

**Published:** 2010-11-17

**Authors:** Stefan A Baeten, N Job A van Exel, Maaike Dirks, Marc A Koopmanschap, Diederik WJ Dippel, Louis W Niessen

**Affiliations:** 1 Netherlands Institute for Health Sciences, PO Box 2040, 3000 CA Rotterdam, The Netherlands; 2 Erasmus University, Department of Health Policy and Management (iBMG), PO Box 1738, 3000 DR Rotterdam, The Netherlands; 3 Erasmus University, Institute for Medical Technology Assessment (iMTA), PO Box 1738, 3000 DR Rotterdam, The Netherlands; 4 Erasmus Medical Centre, Department of Neurology, PO Box 2040, 3000 CA Rotterdam, The Netherlands; 5 School of Medicine, Health Policy and Practice, University of East Anglia, Norwich, UK; 6 Department of International Health, Johns Hopkins School of Public Health, Baltimore, USA

## Abstract

**Background:**

Economic evaluation of stroke services indicates that such services may lead to improved quality of life at affordable cost. The present study assesses lifetime health impact and cost consequences of stroke in an integrated service setting.

**Methods:**

The EDISSE study is a prospective non-randomized controlled cluster trial that compared stroke services (n = 151 patients) to usual care (n = 187 patients). Health status and cost trial-data were entered in multi-dimensional stroke life-tables. The tables distinguish four levels of disability which are defined by the modified Rankin scale. Quality-of-life scores (EuroQoL-5D), transition and survival probabilities are based on concurrent Dutch follow-up studies. Outcomes are quality-adjusted life years lived and lifetime medical cost by disability category. An economic analysis compares outcomes from a successful stroke service to usual care, by bootstrapping individual costs and effects data from patients in each arm.

**Results:**

Lifetime costs and QALYs after stroke depend on age-of-onset of first-ever stroke. Lifetime QALYs after stroke are 2.42 (90% CI - 0.49 - 2.75) for male patients in usual care and 2.75 (-0.61; 6.26) for females. Lifetime costs for men in the usual care setting are €39,335 (15,951; 79,837) and €42,944 (14,081; 95,944) for women. A comparison with the stroke service results in an ICER of €11,685 saved per QALY gained (€14,211 and €7,745 for men and women respectively). This stroke service is with 90% certainty cost-effective.

**Conclusions:**

Our analysis shows the potential of large health benefits and cost savings of stroke services, taking a lifetime perspective, also in other European settings.

## Background

In The Netherlands, as in most Western countries, stroke is a major contributor to the total burden of disease, in terms of morbidity, mortality and concomitant costs. In 2007, incidence of primary stroke was 2.12 per 1,000 men and 2.23 per 1,000 women, prevalence of stroke was 11.89 per 1,000 men and 11.48 per 1,000 women, and mortality from stroke was 46.50 per 100,000 men and 69.84 per 100,000 women [1]. The incidence rates in the Netherlands have only fluctuated around the same level since the early 1990 s. Yet, mortality has been steadily declining [1].

The burden of stroke in the Netherlands is comparable to that in other Western countries [2]. As a result, in 2005 stroke was a top-5 disease in terms of costs, with a total of 1.5 billion Euros. This accounts for 2.2% of total health care costs in the Netherlands [3].

The total burden of disease from stroke is expected to increase. In 20 years, the prevalence of stroke in the Netherlands will be more than 40% higher as a result of aging of the population, continuing unhealthy lifestyles among elderly, and improved care for stroke patients leading to lower mortality [1]. Several studies investigated future trends in life expectancy and disability after stroke in the Netherlands. Struijs et al. [4], used a dynamic single-state life-table combining demographic projections and existing stroke incidence and mortality data, and projected a 30% rise in life years lost between 2000 and 2020. Niessen et al. [5] estimated future stroke morbidity rates using a disability-based two-state transition model combining population projections and existing data on stroke epidemiology. Their projections indicated that the aging of the population and the increase in cardiovascular survival would partially mitigate the effect of the declining incidence on the total burden of stroke, leading to a further increase in major stroke prevalence among the oldest age groups.

An important contribution to increased survival rates after stroke is provided by better coordinated care, including rehabilitation and treatment of complications, through widespread implementation of stroke units, as recommended by the American Heart Association. Integrated stroke services are expected to enhance the early state effect of stroke unit treatment. We characterise integrated stroke services as formal arrangements and strict coordination between various providers of stroke care, with the aim to "provide the right care, to the right patient at the right time". Stroke Services are multi-facetted and need different adaptations in different regional settings. It may be difficult to determine which aspects of stroke services lead to the reported better health outcomes [6-8]. Notable elements of stroke services are: protocolised care, early rehabilitation, prevention of early complications, early supported discharge and secondary prevention. Positive health effects have not only been reported for stroke services as a whole, but also for each of these elements separately [6-9].

Reports on stroke services are more ambiguous on changes in costs [10-14]. The services tend to be cost-effective on the short term compared to traditional care. The short horizon of these findings complicates the formulation of clear recommendations on stroke services. The risks of disability associated with stroke can be high and the chances of new cardiovascular events, stroke or other, are high. Longer survival and these negative health effects may be associated with considerable health care costs. It is unclear whether the positive health effects and potential cost savings will persist in the long run. Consequently, additional evidence on the relative cost-effectiveness of stroke services is needed for longer time horizons.

To our knowledge no long-term follow-up study has reported the cost-effectiveness of stroke services. Long-term results do exist for stroke unit care in the United Kingdom. Using a stratified Markov model Saka et al. showed that stroke unit care combined with early supported discharge provided better health at acceptable costs up to ten years after stroke [15]. In addition, the Department of Health reported cost-effective results for stroke units and early supported discharge, again with a ten year time horizon [16]. However, these results apply to early stage stroke services and do not incorporate continuity of care outside the hospital.

In summary, current evidence shows stroke services to be attractive, yet little is known about the effect of stroke service implementation on long-term mortality, disability and costs. The purpose of the present study is to examine the lifetime cost-effectiveness of stroke services as compared to conventional stroke care, using a life-table approach, differentiating four post-stroke disability categories, assuming persisting health effects. This has required an increase of the disability categories applied in our earlier multi-dimensional life-table study, also used in the evaluation of stroke guidelines [5,17].

## Methods

### Selection of patients

The study used a selection of data from a recent empirical cohort in the Netherlands, the EDISSE study (Evaluation of Dutch Integrated Stroke Service Experiments) [14]. The trial was approved by all participating institutions' ethics committees which was documented in the trial registration (ISRCTN67636203). This prospective non-randomized controlled cluster trial assessed the cost-effectiveness of three stroke service experiments between 1999 and 2000 compared to conventional stroke care in the Netherlands. A stroke service was defined as an integration of a hospital stroke unit with nursing homes, rehabilitation centres, GP's and home care providers to provide adequate services in all stages of the follow-up process [14].

The three trial regions represented the full variety of stroke service care in The Netherlands. Trial and control regions were comparable in terms of case mix; their selection was based on similarity with national stroke statistics in terms of age, length of hospital stay, case-fatality, functional status at discharge, and destination after discharge. The research populations compare well to the demographic profile of the Netherlands. Trained nurses collected data from medical files in hospital and through follow-up patient/proxy interviews two and six months after stroke. Reliability and internal validity were guaranteed by reassessment of files by colleagues or neurologists.

### Intervention contrast

Here, data from one of the three experiments, a stroke service in Delft, was compared to data from all three control regions. This stroke service was a collaboration of a single hospital with an integrated stroke unit; a nursing home with capacity for all diagnosed patients to be admitted and a home care organization with specially trained nurses for stroke patients. In addition, the three organizations made formal mutual agreements about patient flows and continuity of care. Furthermore, home care nurses received additional training and a transmural stroke nurse was in charge of patient transfers. This was the only fully integrated stroke service as defined ex ante, and was the only one cost-effective in the first six months after stroke in the EDISSE trial, while the other did not comply to these criteria and showed indifferent results [14].

The control settings reflected the usual stroke care in The Netherlands at the time (e.g. concerning case load, length of stay and extent of illness). In some settings stroke units were already (being) developed at different care locations in the region, both in hospitals and in rehabilitation centres but not in nursing homes. However, there were no implemented formal agreements between care providers or regular consultations between stroke care providers.

The care process in stroke services differs in many aspects from usual care. This makes the introduction of stroke service a complex intervention. The effects take place within a 'black box' and it will be difficult to identify the effects of single aspect of the stroke service. Table 1 presents the characteristics of the EDISSE study population.

**Table 1:** Characteristics of the study population

	Stroke service	Usual care
N entire EDISSE population	151	187
N with full six month follow-up	90	114
Age	72	73
Women	43 (48%)	65 (57%)
Low educational level (primary school or lower)	34 (38%)	18 (16%)
Living alone at home before stroke	25 (28%)	40 (35%)
Previous stroke	30 (33%)	33 (29%)
Lowered level of consciousness according to Glasgow Coma Scale	3 (3%)	4 (4%)
Haemorrhagic stroke	8(9%)	10 (9%)
Cardiovascular co-morbidity	60 (67%)	66 (58%)
Barthel score at admission:		
Means (SD)	10.8 (6.17)	9.5 (6.19)
Median (Range)	11.5 (0-20)	9 (0-20)

### Disability-stratified stroke life-table

**Figure 1:**
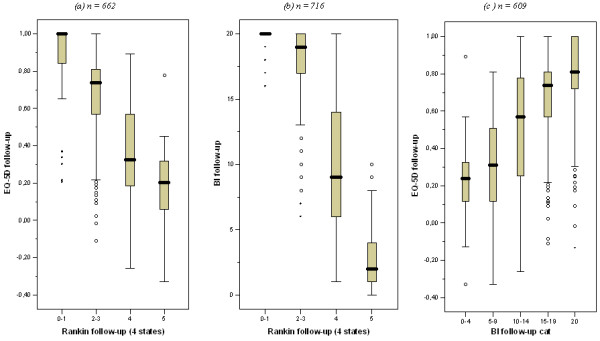
**Relation between EuroQol-5 D, Barthel Index, and modified Rankin Scale (mRS) (4 categories) classification during follow-up**.

A life-table approach was applied to extrapolate the trial findings and to arrive at estimates of lifetime health benefits and costs per patient. A multidimensional Markov structure with four disability categories was adopted, based on the modified Rankin scale (mRS) [17]: category 1 (mRS 0-1); 2 (mRS 2-3); 3 (mRS 4) and 4 (mRS 5). Between these categories significant differences in quality of life exist (see figure 1a). These EQ-5 D ranges are mutually exclusive and show that the two additional disability categories allow for better measurement of health effects than in the original model. Like with EuroQoL-5 D scores, the mRS was not administered at baseline, and Barthel scores were used to classify patients into the four stroke disability categories at baseline (see Figure 1b): Barthel scores 20; 14-19; 5-13 and 0-4 were assigned to category 1, 2, 3 and 4 respectively. This mapping scheme resulted in the least misclassified patients after two and six months of follow-up.

In the multidimensional life-table, patients can move between disability categories depending on the outcomes of stroke recurrence and recovery (see Additional file 1). Patient flows between the four disability categories were based on various epidemiological estimates (see Table 2). Patients exit the life-table only when they die or reach the age of 100. We divided deaths into four categories, each with its own age-specific rates. Clearly, deaths occur because of the stroke itself either (i) immediately after stroke or (ii) from its complications in a later stage. Since stroke patients face higher probabilities of cardiovascular events other than stroke, we modelled (iii) deaths resulting from other cardiovascular events separately. Finally, patients can leave the model because of any other, (iv) non-related cause of death. All death, incidence and recurrences rates are stroke severity specific and based either directly on original epidemiological data or are adjusted through a hazard ratio (see table 2). All transitions are assumed to take place at the end of each cycle of six months. In our life time perspective a half cycle correction is unnecessary as the effects hardly influence the life-time results. All parameters - i.e., the risk of recurrence, case-fatality rate of stroke, probability of disability after stroke, and the four probabilities of death - affect patients' courses in the same way as they did in the original life-table model [5]. The annual probability of a vascular event was assumed constant over time [18].

**Table 2:** Estimates for the disability-stratified stroke-simulation model

Parameter		Unit	Data source	Value*
*Epidemiological measure:*				
Age distribution incident strokes		Rate	Jager [34]	8.46
All cause mortality		Rate	Statistics Netherlands [35]	33.0
Stroke mortality		Rate		1.9
Cardiovascular mortality		Rate		8.4
Death from stroke		Ratio	Herman [36]/Bamford [37]	0.21
Recurrent after mRS 0-3		Relative risk	Hoogen [38]/Dennis [39]/Dutch TIA Trial [40]	
first year	< 75 years			0.09
	> 75 years			
subsequent years				0.05
Late death from mRS 4-5		Ratio	Howard [41,42]	0.15
Death from cardiac disease	after mRS 0-3 stroke	Relative risk	Dutch TIA Trial[40]/Howard [41,42]	0.038
	after mRS 4-5 stroke	Relative risk	Howard [41,42]	0.06
Utility weights for stroke disability categories		-	EDISSE data [14]	0-1
Disability after stroke (first-ever and recurrent)		mRS		1-5
			
*Hazard ratios:*				
Excess cardiovascular death	mRS 0-1:mRS 2-3	Hazard ratio	LiLAC study Group [18]	1.25^†^
	mRS 4:mRS 5	Hazard ratio		1.25
Recurrent stroke	mRS 0-1:mRS 2-3	Hazard ratio		1.34
	mRS 4:mRS 5	Hazard ratio		1.34

Note: * number per 1,000 or probability for men aged 70-75. ^† ^The ratio reported in the Lilac study concerned all vascular events. We assume that the hazard ratio is equal for both cardiovascular event and other vascular events.

The life-table was written in Microsoft Excel, and had the following sequence of calculations: (i) transition probabilities; (ii) number of patients in each disability category and all events (i.e., transitions); (iii) average survival time/life expectancy; (iv) quality-adjusted life expectancy after stroke, defined as the number of survival years multiplied with individual utility values from the EDISSE trial.

### Selection of outcome measures

The economic evaluation compares lifetime health effects and costs of the stroke service as compared to usual stroke care.

#### Health effects

Lifetime health effects were assessed as quality-adjusted life years (QALYs) after stroke, measured with the EuroQoL-5 D [19]. Cost-effectiveness evaluations that take a societal perspective make use of general public valuations of these health states, available from Dutch research [20]. EuroQol-5 D scores were rescaled, using tariff scores, so that the maximum value of 1 represents perfect health and the value 0 represents death; some health states receive a value lower than 0, and are thus considered worse than death by the general public. The EuroQoL-5 D is short and simple enough so that most stroke survivors, despite disabilities, can complete it without help [19]. However, most are physically or mentally not able to (self-) report quality of life in the acute phase after stroke. Therefore, no EuroQoL-5 D scores were available from the EDISSE study at baseline. As in a former study [14,21], scores on the Barthel Index (BI) [22] were used to estimate EuroQoL-5 D scores at baseline to ascertain that quality of life was measured at the acute phase, i.e. the first six months (see Figure 1c). Based on linear regression analysis, health-related quality of life was -0.25 for patients with BI score 0, and increased by 0.05 with each additional BI point. Independent patients (BI score 20) get a health-related quality of life equal to 0.75 [21].

#### Costs

Lifetime costs after stroke were restricted to direct medical costs (i.e., a health care perspective), and computed separately for the four disability categories (see below). This excludes productivity costs as the strokes occurred in elderly patients. We considered the impact on informal care in this study elsewhere [14]. Costs of care for the first six months after stroke were based on patient level resource use from the EDISSE study [13], and resource costs/prices of 2003 [23]. Because length of stay at different locations was the most important cost driver during this period [13], first, inpatient costs were calculated using original length of stay data and 2003 nursing day prices. Subsequently, total individual costs of care during the first six months were computed by holding the original ratio between inpatient and total costs constant, for each patient and each place of residence (hospital, nursing home, revalidation centre and home) at which the patient stayed during this period, weighted by the length of stay. Costs of care for the second half year after stroke were based on place of residence data six months after stroke from the EDISSE study and 2003 resource costs/prices. Costs in subsequent years were based on available data on the distribution of patients by residence location [24]. Therefore, after the first half year, costs are assumed not to differ between stroke service and usual care. In accordance with guidelines, differential discounting was applied with an annual rate of 1.5% for health effects an 4.0% for costs of care. This accounts for the increasing value of health over time. Equal discount rates for costs and health effects lead to sub-optimal societal results [25].

### Cost-effectiveness analysis

The stroke service was compared to usual care by doing the same lifetime extrapolation for both groups, simultaneously. Patient level data (i.e., level of initial stroke disability, costs, and health effects) were entered in a probabilistic analysis, using a Microsoft Excel add-in: Palisade's @Risk. The runs were executed by a bootstrap from the stroke service data. In each iteration, a patient from the usual care data set was matched with the one selected from the intervention region, according to age and level of initial stroke disability. Stroke patient entered the life-table at age 60, 70 or 80, based on the known age distribution of first-ever stroke occurrence. The runs resulted in estimates of lifetime health outcomes (QALYs) and lifetime costs (Euros) in both arms.

Lifetime differences in costs and health effects were compared by means of an incremental cost-effectiveness ratio (ICER) of stroke service care as compared to usual care, i.e., the difference in costs between the two settings divided by the difference in effect. Incremental costs and health effects were plotted in a cost-effectiveness plane, and confidence intervals (5%, 50%, 90%) were computed around the central point using the life table in 10.000 iterations. Sensitivity analysis was conducted using 3% and 0% discounting rates for both costs and health effects.

## Results

### Lifetime costs and health effects

Table 3 presents the average (half) yearly costs and the EuroQoL-5 D score at discharge and 6 months after stroke, differentiated stroke disability level, care setting and gender, which were used to estimate lifetime costs and health effects. These results show that patients treated in the experimental setting were on average in better health. The first six months after stroke showed costs reductions. Highest reductions showed inside the hospital. Here costs are reduced from €10,018 to €5,777, on average. This confirms earlier findings on the reduced length of stay [13].

**Table 3:** Average costs and EuroQoL-5 D score by follow-up period, stroke disability, care setting and gender

		Care setting	mRS 0-1	mRS 1-2	mRS 4	mRS 5	Stroke service (mean)*	Usual care (mean)*
*Number (%)*	At hospital discharge	Stroke service	28 (25)	40 (35)	31 (27)	15 (13)	-	-
		Usual care	14 (16)	40 (44)	29 (32)	7 (8)	-	-
	Six months after hospital discharge	Stroke service	22 (14)	42 (66)	23 (15)	3 (5)	-	-
		Usual care	16 (24)	75 (47)	17 (26)	6 (3)	-	-
								
*Costs (in Euros, 2003)*	0-6 months (including hospital care)	Stroke service	8,400	11,080	29,664	27,371	21.665	-
		Usual care	9,856	14,868	37,628	46,089	-	24.837
	0-6 months (excluding hospital care)	Stroke service	3,434	5,805	23,007	20,428	15.888	-
		Usual care	3,181	6,603	22,430	21,930	-	14.819
	7-12 months	Both	1,761	4,196	17,824	22,515	9.633	9.826
	after 1 year (men, 6-monthly costs)	Both	811	1,028	5,997	7,633	3.109	3.233
	after 1 year (women, 6-monthly costs)	Both	811	1,028	9,900	12,702	4.761	4.990
*Health-related quality of life*	*at discharge*	Stroke service	0.7500	0.6245	0.1667	-0.1739	0.3701	-
		Usual care	0.7500	0.6163	0.2238	-0.1413	-	0.4201
	*after 6 months*	Stroke service	0.8979	0.7726	0.6758	0.3030	0.7111	-
		Usual care	0.8233	0.6863	0.5351	0.2371	-	0.6239

Note: * Weighted average over four stroke disability categories

**Figure 2:**
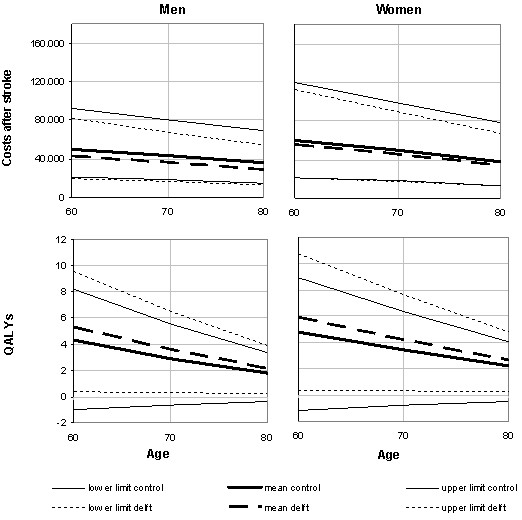
**Lifetime costs (in Euros) and health-related quality of life after stroke; mean values (90% CI) by age and gender**.

Life expectancy after stroke of patients in mRS categories 0-1, 2-3, 4 and 5 was 5.94, 5.16, 3.87 and 3.64 for men, and 6.91, 5.94, 4.92, and 4.51 for women. Corresponding lifetime QALYs were estimated at 4.12, 3.00, 1.39, -.02 for men, and 4.80, 3.28, 1.69, -0.01 for women, respectively. Figure 2 shows that lifetime costs and quality-adjusted life expectancy decrease with age, both among men and women. Furthermore, the figure shows that stroke services likely are cost saving, while generating more QALYs. The lower costs in stroke services resulted from shorter mean length of stay in hospital in the acute phase after stroke (13 vs. 29 days [13]), and the lower proportion of patients who were institutionalized one year after stroke (14% vs. 23%).

The overall life time costs for men were € 32,284 in the stroke service setting and € 39,335 in the control setting while the life time QALYs were 2.92 and 2.42 respectively. For women both costs (€ 38,443 in the stroke service and € 42,944 in the usual care) while the life time QALYs were higher (3.33 and 2.75 years). Standardized for gender the results for the stroke service were average costs of € 35.361 and 3.12 QALYs and for the usual care setting €41,352 and 2.61 QALYs. So, in all three cases stroke services are associated with lower costs and higher life time QALYs, i.e. stroke services dominate usual care.

### Cost-effectiveness results

**Figure 3:**
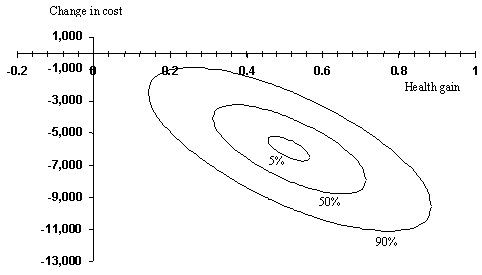
**Cost-effectiveness plane; reliability intervals (5%, 50%, 90%) for lifetime cost-effectiveness of stroke services as compared to usual stroke care**.

Figure 3 presents the reliability intervals for the lifetime cost-effectiveness of the stroke service as compared to usual care, with a central point representing a cost saving of € 5,990 and a QALY gain of 0.51. The point estimate for the ICER is €11,685 saved per QALY gained; €14,211 and €7,745 saved per QALY gained for men and women respectively. The probability that the stroke service intervention is both effective and cost saving is over 90%. The ICER declines with age. In addition, Figure 3 shows a negative correlation between health effects and costs as lower health care consumption and better health are associated.

Discounting both costs and health effects at a 3% rate lead to slightly lower ICERs of € 15,510 and € 8,423 saved per QALY gained for men and women, respectively. Discounting both at 0% lead to ICER estimates of € 14,144 and € 7,401 saved per QALY gained for men and women, respectively. The results therefore showed robustness and consistency in all age specific outcomes: mean health effects were larger and mean costs were lower in the stroke service in all age groups, both genders, and with all three ways of discounting.

## Discussion

Our analysis presented mRS-stratified lifetime costs and health effects after stroke and showed that stroke service interventions most likely will lead to health benefits and cost savings when considering lifetime outcomes. Our results imply that the beneficial short term effect of stroke services is not offset by a long term costs of treatment and care because of longer survival.

The estimated health gain from stroke service implementation is substantial (about half a QALY), especially as compared to the total number of QALYs usually lived after a stroke (on average 2.42 for men and 3.33 for women in usual care). The estimated lifetime cost savings of € 5,776 (14%) from stroke service implementation are also substantial. Although we did not incorporate start up and nationwide implementation costs, the figures compare very well to recent figures by Struijs et al. [4], who estimated that a nationwide implementation of stroke services in the Netherlands would result in a 13% reduction of the costs of stroke as compared with a regular care scenario.

The lifetime cost-effectiveness of stroke service implementation is comparable to the short-term (first 6 months after stroke) results presented by us in an earlier study using the same trial data [14]. While the "lifetime ICER" was € 11,685 saved per QALY gained, with a 90% likelihood of the stroke service being cost saving. The "short-term" ICER was € 19,350 saved per QALY gained, with an 80% likelihood of the stroke service being lower than €.35,000 per QALY gained. Our lifetime outcomes after stroke show less cost savings but do therefore provide further support that stroke services are the organisation of choice as compared to usual fragmented care.

Stroke unit reviews use other outcome measures than ICERs. The Cochrane library nor the HEED data base report reviews of stroke unit cost-effectiveness evaluations, but the reviews on their effectiveness is strong [26]. The results of other studies ranged from a reduction of a relative risk of dependency for stroke patients of 9% [27], to a 28 week cost reduction of € 567 [11]. Two cost-effectiveness studies showed results ranging from 16,790 €/QALY gained [28] in the Netherlands to 90,699 €/QALY gained (64,097 £/QALY gained) [12] in the UK. The latter study [12] compared three alternative strategies but lacked a comparison with usual care.

We have made some critical choices in our evaluation. The EDISSE study included three experimental and three usual care settings. We analyzed data on all three usual care settings, but limited ourselves to the only real-life experiment that implemented a stroke service completely according to national guidelines [14]. So, our results are only valid for similar settings (a single hospital with supporting follow-up services). Our results may therefore be optimistic and limit the options for wide implementation as there are many settings with more hospitals and a diversity of stroke rehabilitation services. We would have included the (negative) results from the other two settings only if the service model would have turned out similar.

Next, we have not included indirect cost. Earlier [13] we showed that there was an increase in home-based and ambulatory health care cost (through professional support and increased revalidation efforts). It might be that the service set-up leads to additional cost of informal care and patients are discharged earlier. We cannot confirm that this is not happening. In other studies [29] it is explained that these cost are relatively low, also after valuation. It is our expectation that these would not alter the conclusion of our cost-effectiveness analysis, also when taking this societal perspective.

Some comments are to be made on the stroke model life tables. First, our model synthesizes data from different studies and settings (see Table 2), giving a population level estimates of the stroke burden. This approach is similar to two recent UK studies [15,16] with all three studies arriving at comparable conclusions (although the latter applies only a ten year time horizon). Data on mortality, hospitalization, and nursing home admission rates were available from existing studies. The hazard ratios for recurrent stroke and excess cardiovascular death, however, were only available for patients with a score on the mRS of 3 or lower. In the model, the same hazard ratios were used to differentiate between mRS 4 and mRS 5 patients as between mRS 0-1 and mRS 2-3 patients. These assumptions do not influence the cost-effectiveness results.

Second, the classification of patients at baseline was based on the mapping of Barthel Index scores on Rankin scale categories, because the mRS is less reliable in the acute clinical phase after stroke. Although the correlation between the measures was large, the chosen procedure may have led to some misclassification of patients, resulting in higher uncertainty in survival outcomes.

Third, the bootstrap conducted for each age group and gender was based on the same patients. Patients were not entered by age and gender due to sample size limitations, which means that it was assumed that the effect of stroke and stroke treatment is independent of age and gender [30]. While it is still unclear how the impact of a stroke on an individual's quality of life varies by age [31,32], this does not mean that quality of survival is equal for different ages and gender, as assumed in the model. This is expected to have more effect on the lifetime outcomes differentiated per age and gender, than on total outcomes.

Finally, the results presented here were based on a life-table extrapolation of data originating from studies with sometimes short follow-up periods. For a more accurate estimate of the lifetime cost-effectiveness of stroke services, it would be necessary to conduct similar studies with a much longer follow-up. These do not exist (yet). Nonetheless, the disability stratified model presented here is the most comprehensive and detailed analysis currently available for estimating the lifetime health and costs after stroke for The Netherlands.

Summarizing, this study confirms previous findings that, from a health care perspective, effective coordination between health care providers involved in the rehabilitation of stroke patients, through integrated stroke services, may result in positive lifetime health effects at lower costs. Previous studies described the effects of interventions limited to early stage stroke units. Our study included the additional long-term health effect and organization effects associated with extra coordination between different health care organizations opposed to coordination within a single organization.

Our findings support the recommendations of the European Stroke Initiative to provide disabled stroke patients with early institutionalized rehabilitation by a multidisciplinary team [33]. However, length of stay in stroke units may vary within Europe and this may change the financial, short-term, impact of stroke services outside the Netherlands. Likewise, case load and severity may be dissimilar in different settings. Unlike in many European countries treatment for stroke patients in specialized hospital units is common in the Netherlands. The intervention effect measured in this study, taking the Delft example, is very likely an underestimation of the potential much larger impact of stroke service introduction in countries without specialized hospital stroke care. In sum, although transferability of stroke services set-ups to different setting needs to be accounted for, both in health and economic terms, we do recommend implementation of stroke services in a wider array of country settings.

## Competing interests

The authors declare that they have no competing interests.

## Authors' contributions

SB: Developed the model, performed the analysis and drafted the manuscript. JvE provided data and details on the EDISSE study and edited the draft manuscript. MD helped with the clinical aspects of the model and effectiveness results. MK assisted in the calculation of health care costs and provided details on the EDISSE study. DD participated in the study design and helped with the clinical interpretation of the model and its results. LN was responsible for the study design, coordination, model development and edited the drafted manuscript. All authors read and approved the final manuscript.

## Supplementary Material

Additional file 1 **Life-table equations**. Additional file 1 describes the generic equations used in the disease model for the calculation of the transition probabilities between disease states. It also describes the equation used to estimate the life-tables outcomes, i.e. average quality of life and average health care costs. Click here for file
