# Supplementary material for: Lifetime health effects and medical costs of integrated stroke services - a non-randomized controlled cluster-trial based life table approach
Source: Cost Eff Resour Alloc. 2010 Nov 17;8:21. doi: 10.1186/1478-7547-8-21 (PMC2998455; doi:10.1186/1478-7547-8-21)
Supplement: Additional file 1 — Life-table equations. Additional file 1 describes the generic equations used in the disease model for the calculation of the transition probabilities between disease states. It also describes the equation used to estimate the life-tables outcomes, i.e. average quality of life and average health care costs. [file 1478-7547-8-21-S1.PDF]

Life-table equations

Transition matrix

At each time interval the distribution of patients over the disability categories changes through a transition matrix A_0*: A fraction* $\lambda_{i,j}$ *(i=1...4 and j=1…4)* moves to a more disability category, while other remain in the same category, except in the worst mRS5 category in which people can only die.

$$A_0 = \begin{bmatrix} 1-\lambda_{1,2}-\lambda_{1,3}-\lambda_{1,4} & 0 & 0 & 0 \\ \lambda_{1,2} & 1-\lambda_{2,3}-\lambda_{2,4} & 0 & 0 \\ \lambda_{1,3} & \lambda_{2,3} & 1-\lambda_{3,4} & 0 \\ \lambda_{1,4} & \lambda_{2,4} & \lambda_{3,4} & 1 \end{bmatrix}$$

Here, i and j represent the disability categories mRS 0-1 through – mRS 5. The disability-stratification is also reflected in the columns (i), which is the disability category at period t and the rows (j) which is the disability category in period t+1.

Quality of life and costs equations

Average quality adjusted life expectancy per 1,000 patients

$$= \frac{\sum_{mRS=0}^{6} \sum_{t=60}^{t=100} EQ_{mRS,t} * N_{mRs,t}}{1000}$$

Average lifetime costs after stroke per 1,000 patients

$$= \frac{\sum_{mRS=0}^{6} \sum_{t=60}^{t=100} EQ_{mRS,t} * C_{mRs,t}}{1000}$$

Where: mRS = stroke disability category

t = age patient

EQ = EuroQol-5D utility weight

C = medical costs

N = 1000. Number of people in the life-table
